# Knowledge, Attitude, Frequency and Level of Consumption Regarding Non-alcoholic Carbonated Soft Drinks among Students from Two High Schools in Hanoi, Vietnam in 2015

**DOI:** 10.3934/publichealth.2017.1.62

**Published:** 2017-02-16

**Authors:** Nguyen Thanh Ha, Le Thi Thu Ha, Luu Quoc Toan

**Affiliations:** Department of Nutrition and Food Safety, Hanoi University of Public Health, 1A Duc Thang street, North Tu Liem District, Hanoi, Viet Nam.

**Keywords:** non-alcoholic carbonated soft drinks, knowledge, attitude, frequency, level of consumption

## Abstract

**Objective:**

This article aims to describe the knowledge, attitude, frequency and level of consumption regarding non-alcoholic carbonated soft drinks (NCSD) among students from two high schools in Hanoi.

**Materials and Methods:**

A cross-sectional survey including a semi-quantitative food frequency were conducted with 620 students from two high schools, one in the urban area and the other in the rural area of Hanoi city.

**Results:**

Data on knowledge of health risk associated with the consumption of NCSD showed neagtive results (only 11.9% of the students were able to identify all the contents of NCSD correctly, and 2.7% knew all eight health risks due to consumption of NCSD). Besides, 31.4% of all students did not have the intention to quit NCSD despite being aware of health risks associated with the consumption of NCSD. Students who reported consuming NCSD within one month prior to the study constituted 83.1%, and those who consumed NCSD 1–2 times/week accounted for the highest proportion, being 21.3%. On average, each student consumed 2,094 ml NCSD within one month prior to the study. Suburban students and male students consumed more than urban and female ones, respectively (*p* < 0.01).

**Recommendations:**

Students should be equipped with information about NCSD related health risks and encouraged to consume less NCSD.

## 1. Introduction

The consumption of NCSD is prevalent among youngsters worldwide [1],[2]. NCSD contain saturated carbon dioxide, sweeteners, and other ingredients such as flavor enhancers, salts, additives and preservatives [3]. In 1997, each person in the world consumed approximately 36 liters of NCSD a year, and the consumption level increased to 43 liters in 2010 [4]. According to a report on the trend of NCSD consumption among Americans in 2012, American families consumed three types of NCSD a week, and 62% of American adults drank at least one type of NCSD every two weeks. Besides, 49% of the NCSD was consumed at lunch while 31% at dinner [5]. A study in Australia indicated that the daily amount of NCSD consumed per capita increased by age. Particularly, children aged 2–3 years drank 53 ml as opposed to 364 ml consumed by those aged 16–18 years. In the latter group, a male drank 480 ml compared to 240 ml by a female [6].

Several studies gave warnings about harmful effects of excess daily NCSD consumption on health such as overweight, blood lipid disorders, diabetes, gout and cancer [4],[6]–[8]. Daily consumption of NCSD can increase the risk of overweight in both children and adults [4],[9],[10]. Children aged 11–13 years who drank fewer than three cans of NCSD a day had a 1.02 times lower BMI z-score (adjusted for age and gender) compared to those consuming three or more cans of NCSD a day [11]. Besides, certain studies reported low level of knowledge and attitude regarding NCSD. One study carried out in 2013 with 110 second-year medical students in India showed that only six students (5.5%) were able to identify all ingredients of NCSD correctly. Nearly eighty students (73%) had good knowledge of adverse effects of NCSD, and 69 students (62.7%) reported never having tried to give up the habit of consuming NCSD [12]. A study on KAP related to NCSD consumption among students aged 8–17 years in India revealed that as many as 17.1% from group I (lower socio-economic group) and 28.3% from group II (higher socio-economic group) thought that NCSD caused no harm to oral health [13].

In Vietnam NCSD consumption at festivals, celebrations or meetings is very common with a consumption rate of 75.8%. The proportion of people consuming NCSD 3–4 times/week and 1–2 times/week were 28.6%, and males drank NCSD more frequently than females [14]. The levels of NCSD consumption in Vietnam are identified mainly on the basis of the total volume of NCSD consumed through out the country. Besides, very few studies in Vietnam explored the knowledge and attitude regarding NCSD consumption, especially among high students who are more likely to consume such drinks than any other group in the general population [3],[14],[15]. This article aimed to describe the knowledge and attitude of students fromtwo high schools in Hanoi in 2015 and identify their frequency and level of NCSD consumption within one month prior to the study.

## 2. Materials and Methods

### 2.1. Study Design

This descriptive cross-sectional study using face-to-face interviews and a semi-quantitative food frequency questionnaire was conducted between January 2015 and August 2015, and data collection was carried out in the end of April 2015.

### 2.2. Sampling

The minimum sample size calculated for this study was 645 students. The sample size was calculated based on the formular used for estimation of a proportion with a specifed precision. The values of the parameters for sample size calculation were as follows: Z_1-α/2_ = 1.96 for 95% CI (α = 0.05); estimated population proportion of those who have good knowledge of NCSD consumption (*p* = 0.273) [12], design effect allowing for multistage sampling (DE = 2), and margin or error (d = 5%). This sample size also allowed for a non-response rate of 5%. With this sample size, the students needed to be recruited in each school ranged from 320 to 325.

Multi-stage sampling was applied in this study. Firstly, two high schools, one in the urban area and the other in the suburban area of Hanoi, were randomly selected from a list of all high schools in each area. Each class from those schools had between 50 and 55 students; hence, six classes, including two 10th grade classes, two 11th grade and two from 12th grade, were randomly selected by drawing from either school. All students of the six selected classes were recruited to the study. However, in fact, only 620 students from two schools participated in the study.

### 2.3. Data Collection

At the end of each school day, students had their weights and heights measurd using SECA electronic scales with an accuracy of 100 grams and UNICEF's wooden three-piece height boards with an accuracy of 0.1 cm. After that, in order to collect data about the students' knowledge and attitude regarding NCSD, they were interviewed using a questionnaire that had been piloted with 15 students and then revised carefully.

Data about the frequency and level of consumption of NCSD within one month prior to the study were collected by using a semi-quantitative food frequency questionnaire which had been piloted with 15 students and revised so that the questionnaire was understandable to the participants. All types of NCSD commonly available in the local market at the time of the study were listed in the questionnaire, and the investigators asked the students how many times they consumed each type of NCSD daily, weekly and monthly and on special occasions such as festivals, celebrations, or meetings within the previous month and the amounts of NCSD they consumed each time. In order to minimize recall biases, the interviewers reminded the students of important events in which they were likely to consume NCSD within the month prior to the study, including holidays, festivals, or birthday parties. Besides, the interviewers showed the students pictures of glasses, cans and bottles commonly used as containers of the NCSD to help the students estimate more exactly the amount of NCSD they consumed on each occasion, measured in mililiter.

By the end of each day, all questionnaires were cross-checked in order to identify and timely recollect missing information.

### 2.4. Measures

#### 2.4.1. Outcome Variables

Students' knowledge of NCSD was measured based on their understanding about ingredients of the NCSD, the benefits of NCSD and health risks associated with NCSD consumption.

Students' attitude toward NCSD was measured based on how much they agree with NCSD consumption and how willing they were to stop consuming NCSD if being aware of health risks.

The frequency of NCSD consumption was categorized into 8 levels: 1–2 times/week, 3–5 times/week, 6–7 times/week, 1–2 times/month, 3–5 times/month, 6–7 times/month, only on the special occasions (e.g. festivals, celebrations, or birthday parties), and rarely.

The amount of NCSD consumed within one month prior to the study was calculated by total amount of NCDS consumption of each student within one month prior to the study.

#### 2.4.2. Explanatory Variables

The background information about the study participants included age, gender, grade, weight, height, BMI, nutritional status, perceived household economic status, and fathers' and mothers' highest education levels.

The nutritional status of the students was categorized into three levels, namely malnourished (BMI for age < -2SD); normal (-2SD ≤ BMI for age ≤ +1SD); and overweight (BMI for age > +1SD).

Perceived household economy was self assessment by student and categorized into four levels, namely high income, middle income, low income, and don't know/don't answer.

Parents' education levels were reported by the students and categorized into two levels, namely less than high school, and high school and above.

### 2.5. Data Analysis

Data were entered into Epi data 3.0, randomly checked to ensure their accuracy and analyzed using SPSS 19.0. The knowledge, attitude and frequencies regarding NCSD consumption were presented in tables in the form of frequencies and proportions. Mean values were used to calculate the average amount of NCSD consumed within one month prior to the study.

### 2.6. Ethical Considerations

The study was approved by the Ethical Committee of Hanoi School of Pubic Health. The research team obtained approval to conduct the study from the Boards of Management of two high schools before the data collection was undertaken. In order to maximize the participation rate, the interviewers explained clearly to the students about the study objectives, the study procedures and informed them that participation in the study was completely voluntary, which meant that they could withdraw from the study at any time without any consequences. Each student was given a consent form and asked to read it carefully and sign it before the interview started. At the end of each, a couselling session about possible health risks associated with NCSD consumption was provided to the student by the research team. The study results were to be disseminated to relevant stakeholders in order to inform policies and interventions to improve the health of young people and paved the way for future studies.

## 3. Results

In total 620 students from two schools participated in the study. The study population consisted 43.5% males and 56.5% females; this ratio was relatively conistent among 10th, 11th and 12th grades. Most students (77.6%) thought their households had a middle-level income, and urban students from middle-income households out numbered those from suburban areas (81.7% vs. 73.6%). The mean weight of male students was 57.1 kg compared to 48.3 kg in female students. Male students from the urban area had greater weight than those from the suburban area, and this was also true in the case of female students (*p* < 0.05). Malnourished students accounted for 5.2%, and students from both schools who suffered from overweight and obesity made up 7.7%; the rate of overweight and obesity in urban area was three times higher than that in the suburban one (11.9% vs. 3.9%) (*p* < 0.05). The rate of parents in the urban area who had education level of high school and above exceeded that of those in the suburban area (*p* > 0.05). Detailed information is presented in Table 1.

**Table 1.** General information about high school students.

Contents	Details	Urban n = 313	Suburban n = 307	Total n = 620
**Grade** (n, %)	Grade 10	116 (37.1)	98 (31.9)	214 (34.5)
	Grade 11	87 (27.8)	110 (35.9)	197 (31.8)
	Grade 12	110 (35.1)	99 (32.2)	209 (33.7)
**Mean Age** (X ± SD)	Male	16.3 ± 1.0	16.5 ± 0.9	16.4 ± 0.9
	Female	16.3 ± 1.0	16.2 ± 0.9	16.3 ± 1.0
**Gender** (n, %)	Male	140 (44.7)	130 (42.3)	270 (43.5)
	Female	173 (55.3)	177 (57.7)	350 (56.5)
**Mean weight**(X ± SD)	Male *	60.1 ± 11.6	53.8 ± 8.1	57.1 ± 10.5
	Female *	50.3 ± 7.8	46.3 ± 5.4	48.3 ± 6.9
**Mean height**(X ± SD)	Male	168.2 ± 5.6	165.7 ± 6.2	167.0 ± 6.0
	Female	156.2 ± 5.5	155.4 ± 4.8	155.8 ± 5.2
	Female	-0.23 ± 0.89	-0.71 ± 0.80	-0.47 ± 0.88
**Nutritional status**	Malnourished	11 (3.5)	21 (6.8)	32 (5.2)
**Father's education level** (n, %)	Normal	266 (85.0)	274 (89.3)	540 (87.1)
	Overweight *	36 (11.5)	12 (3.9)	48 (7.7)
	Less than high school	63 (20.1)	117 (38.1)	180 (29.0)
	High school or above	250 (79.9)	190 (61.9)	440 (71.0)
**Mother's education level** (n, %)	Less than high school	59 (18.8)	121 (39.4)	180 (29.0)
	High school or above	254 (81.2)	186 (60.6)	440 (71.0)
**Perceived household economy** (n, %)	High income	6 (1.9)	12 (3.9)	18 (2.9)
	Middle income	256 (81.5)	226 (73.6)	481 (77.6)
	Low income	32 (10.2)	42 (13.7)	74 (11.9)
	Don't know/Don't answer	20 (6.4)	27 (8.8)	47 (7.6)

*: *p* < 0.05 (comparison between urban and suburban schools).

### 3.1. Knowledge and Attitude Regarding NCSD

The results in Table 2 indicate that the students had very poor knowledge of the ingredients of NCSD; only 11.9% of the students were able to identify all ingredients correctly (9.9% urban students vs. 14.0% suburban students). Three ingredients most commonly reported by students were sugar (78.7%), carbon dioxide (69.2%) and color additives (69.2%). Noticeably, 9% and 5.9% of urban and suburban students knew none of the ingredients of NCSD, respectively.

A relatively high proportion of students misunderstood that NCSD had some benefits for the body. About two thirds of them thought NCSD could help reduce tiredness (71.4%), and 43.7% of the students from both schools considered that NCSD could provide energy for the body. Differences between urban and suburban rates regarding positive effects of NCSD were not statistically significant.

Table 3 also shows that the students had poor knowledge about health risks associated with NCSD consumption, irrespective of which school they came from. Diabetes and flatulence/dyspepsia were the two most commonly reported health risks most commonly (59.7% and 54.8%, respectively). Students who were aware of the health risks associated consumption of NCSD accounted for 14%. Only 2.7% of the students answered all of the eight questions related to health risks correctly.

Table 4 illustrates the students' attitude toward NCSD consumption. Only 41.3% of the students disagreed with NCSD consumption. The disagreement rate among suburban students exceeded that among urban students. Similarly, female students who disagreed with NCSD consumption out numbered male ones (*p* < 0.05).

Despite being aware of the health risks associated with the consumption of NCSD, about one third of students (31.4%) were not willing to stop the habit of drinking them. The proportion of urban students willing to stop drinking NCSD was quite similar to that of those from the suburban area. Likewise, similar proportions were also found among male and female students.

**Table 2.** Knowledge of the ingredients of NCSD.

Ingredients	Urban school n = 313 (n, %)	Suburban school n = 307 (n, %)	Total n = 620 (n, %)
Sweeteners	257 (82.1)	231 (75.2)	488 (78.7)
Carbon dioxide	215 (68.7)	214 (69.7)	429 (69.2)
Alcohol	76 (24.3)	82 (26.7)	158 (25.5)
Flavor enhancer	187 (59.7)	175 (57.0)	362 (58.4)
Additives	148 (47.3)	144 (46.9)	292 (47.1)
Preservatives	204 (65.2)	178 (58.0)	382 (61.6)
Color additives	214 (68.4)	215 (70.0)	429 (69.2)
Others	7 (2.2)	0	7 (1.1)
Don't know	28 (9.0)	18 (5.9)	46 (7.5)
**All ingredients**	**31 (9.9)**	**43 (14.0)**	**74 (11.9)**

**Table 3.** Knowledge about effects of NCSD on human body.

Contents	Urban school n = 313 (n, %)	Suburban school n = 307 (n, %)	Total n = 620 (n, %)
**Misunderstanding about the positive effects of NCSD on human body**			
NCSD help reduce tiredness	217 (69.8)	224 (73.0)	441 (71.4)
NCSD help digestion of food	78 (25.1)	67 (21.8)	145 (23.5)
NCSD provide energy	132 (42.4)	138 (45.0)	270 (43.7)
Others	9 (2.9)	9 (2.9)	18 (2.9)
**Understanding about health risks associated with consumption of NCSD**			
Diabetes	203 (64.9)	167 (54.4)	370 (59.7)
Flatulence/dyspepsia	173 (55.3)	167 (54.4)	340 (54.8)
Cancer	77 (24.6)	92 (30.0)	169 (27.3)
Hyperlipidemia	79 (25.2)	74 (24.1)	153 (24.7)
Overweight-obesity	99 (31.6)	41 (13.4)	140 (22.6)
Micronurtrient deficiency	58 (18.5)	46 (15.0)	104 (16.8)
Osteoporosis	44 (14.1)	47 (15.3)	91 (14.7)
Gout	30 (9.6)	25 (8.1)	55 (8.9)
Don't know	48 (15.3)	39 (12.7)	87 (14.0)
**Answered correctly all of the eightitems relating to health risks**	**11 (3.5)**	**6 (2.0)**	**17 (2.7)**

**Table 4.** Students' attitude toward NCSD consumption.

Contents	Strongly agree (n, %)	Agree (n, %)	Disagree (n, %)
**Level of agreement with NCSD consumption**			
**Area** (n = 620)			
Urban school	40 (12.8)	169 (54.0)	104 (33.3) *
Suburban school	25 (8.2)	130 (42.3)	152 (49.5) *
Total	65 (10.5)	299 (48.2)	256 (41.3)
**Gender** (n = 620)			
Male	45 (16.6)	145 (53.7)	80 (29.6) *
Female	20 (5.7)	154 (44.0)	176 (50.2) *
**Level of willingness to quit the habit of consuming NCSD if being aware of their health risks**			
**Area** (n = 620)			
Urban school	112 (35.7)	105 (33.5)	96 (30.7)
Suburban school	94 (30.6)	114 (37.1)	99 (32.3)
Total	206 (33.2)	219 (35.3)	195 (31.4)
**Gender** (n = 620)			
Male	81 (30.0)	99 (36.7)	90 (33.3)
Female	125 (35.8)	120 (34.3)	105 (30.0)

*: *p* < 0.05, comparison between the urban vs. suburban schools and male vs. female.

### 3.2. Frequency and Level of Consumption of NCSD

**Figure 1.**
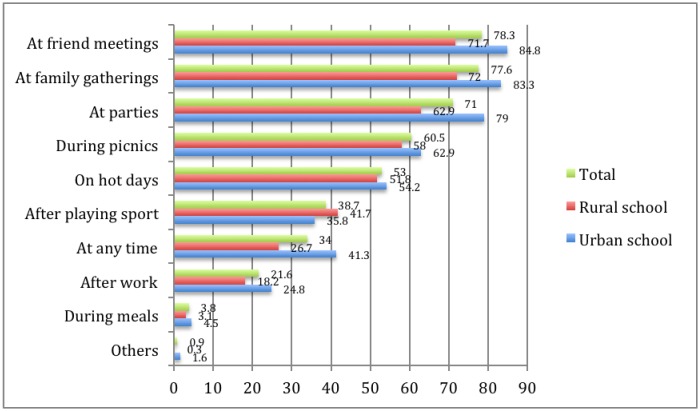
Occasions on which NCSD were consumed (n = 620).

The study results showed that 100% of the students had consumed NCSD before. Figure 1 below illustrates the occasions on which students consumed NCSD.

In a descending order, five most common occasions on which NCSD consumed included friend meetings, family gatherings, parties, picnics and hot days. Noticeably, the rate of drinking NCSD during meals among urban students was higher than that among suburban ones (21.9% vs. 9.4%), and the rate of drinking NCSD at any time among urban students was twice as much as that among suburban students (41.3% vs. 26.7%).

**Table 5.** Frequency of NCSD consumption within one month prior to the study.

Level of consumption	Urban school (n, %)	Suburban school (n, %)	Total (n, %)
Used to consume NCSD (n = 620)	313 (100.0)	307 (100.0)	620 (100.0)
Consumed NCSD within one month prior to the study (n = 620)	260 (83.1)	255 (83.1)	515 (83.1)
Frequency of NCSD consumption within one month prior to the study (n = 515)			
Daily	17 (6.5)	27 (10.6)	44 (8.5)
1–2 times/week	53 (20.4)	58 (22.7)	111 (21.6)
3–5 times/week	42 (16.2)	38 (14.9)	80 (15.5)
6–7 times/week	5 (1.9)	14 (5.5)	19 (3.7)
1–2 times/month	33 (12.7)	31 (12.2)	64 (12.4)
3–5 times/month	29 (11.2)	25 (9.8)	54 (10.5)
6–7 times/month	19 (7.3)	13 (5.1)	32 (6.2)
Only consumed on special occasions (e.g. festivals, celebrations, or birthday parties)	49 (18.8)	43 (16.9)	92 (17.9)
Rarely	13 (5.0)	6 (2.4)	19 (3.7)

The proportions of students from two high schools consuming NCSD within one month prior to the study were exactly the same (83.1%). Students consuming NCSD 1–2 times/week accounted for the highest proportion (21.6%), followed by the proportion of those only drinking NCSD on special occasions (17.0%), and the proportion of those consuming NCSD 3–5 times/week (15.5%). About 8.5% of students reported daily consumption of NCSD.

As can be seen in Table 6, on average, each student consumed 2,094 ml of NCSD or six 330-militer cans every month. The level of NCSD consumption varied between study sites and genders (*p* < 0.05). More specifically, urban students consumed a smaller amount of NCSD than did suburban ones (1,630 ml vs. 2,568 ml) (*p* < 0.05). Male students drank 1.5 times more than female ones (2,833 ml vs. 1,525 ml) (*p* < 0.01).

When looking at NCSD amounts by nutritional status, it was found that malnourished students were more likely to consume NCSD (median = 1,320 ml, and mean = 2,484.4 ml). However, the difference between the nutritional status groups was not statistically significant (*p* > 0.05). Regarding household economy, the amount of NCSD consumed by students from high income households was three times higher than that consumed by the other groups (*p* > 0.05). No difference regarding the amounts consumed were found between students whose parents completed high school or higher level of education and those whose parents did not complete high school.

**Table 6.** The total amount of NCSD consumed within one month prior to the study.

Contents	Details	Median (Min; Max) (ml)	Mean (X ± SD) (ml)	*p*
**Area**	Urban (n = 260)	1,320 (160 ; 9,360)	1,630.5 ± 2,604.3	*p* < 0.05
	Suburban (n = 255)	1,569.3 (110; 7,520)	2,568.4 ± 3,979.1	
	Total (n = 515)	1,300 (110; 9,360)	2,094.9 ± 3,386.1	
**Gender**	Male (n = 243)	1,440 (90 ; 9360)	2,833.6 ± 4,001.5	*p* < 0.01
	Female (n = 272)	1,040 (120 ; 8,910)	1,525.1 ± 2,692.0	
**Nutritional status**	Malnourished (n = 29)	1,320 (110,0; 9,220)	2,484,4 ± 1,744.2	*p* > 0.05
	Normal (n = 448)	995.0 (100,0; 6,600)	2,074.2 ± 2,219.6	
	Overweight and obesity (n = 38)	840.0 (150.0; 9,240)	2,069.0 ± 2,772.0	
**Perceived household economy**	High income (n = 15)	1,215.0 (330; 9,900)	2,299.4 ± 2,052.6	*p* > 0.05
	Middle income (n = 405)	1,000 (100 ; 5,600)	2,014.4 ± 1,099.6	
	Low income (n = 60)	985.0 (170 ; 7,700)	2,173.2 ± 2,248.6	
	Don't know/don't answer (n = 35)	840,0 (110; 9,110)	1,615.4 ± 2,374.7	
**Father's education level**	Less than high school (n = 156)	1,077.5 (110; 7,700)	2,145.8 ± 3,647.6	*p* > 0.05
	High school or above (n = 359)	990.0 (100; 8,630)	2,074.1 ± 3,277.2	
**Mother's education level**	Less than high school (n = 149)	1,025.0 (100; 9,700)	2,030.2 ± 2,279.7	*p* > 0.05
	High school or above (n = 366)	990.0 (100; 8,700)	2,121.4 ± 2,432.0	

## 4. Discussion

Very few epidemiological studies on knowledge and attitude toward NCSD have been done in Vietnam. In review literature relevant to this study, the research team did not find any study on such knowledge and attitude but a small number of reports and articles on the habit of NCSD consumption and NCSD market [3],[14],[15].

### 4.1. Students' Knowledge of NCSD

There have been growing concerns about the negative impacts of NCSD on human health. Our study results showed that students from two high schools had poor knowledge about ingredients of NCSD, and misunderstood about the benefits of NCSD to humanbody and the health risks assoicated with NCSD consumption. Our study results are consistent with those ofa study conducted in 2013 on 110 medical students in India, in which 100% of the participants reported having heard of NCSD, but only 5.5% of them knowing all the contents printed on NCSD bottles. Moreover, 73% of the students in our study knew detrimental effects of NCSD consumption, and 31% said that overweight and obesity were the main consequences of NCSD consumption [12]. The results are also consistent with those of a study that revealed students and adolescents' low level of knowledge about adverse effects of NCSD on dental and oral health [13],[16].

Although NCSD are advertised in the mass media, advertisements seem only to “stimulate” consumers' thirst for purchasing and consuming such drinksand offer attractive promotions to consumers while ignoring health risks associated with NCSD consumption. Advertisements sometimes exagge rate the benefit of NCSD and feature celebrities to make products more appealing to youngsters [11]. Advertising can have long-term impression on young people and influences their habit of consuming NCSD. Given that young people are the biggest group ofNCSD consumers, not many of them have good understanding about such drinks [11]. This means that in this era of information explosion, young people still need to be provided with knowledge about the adverse effects of NCSD through communication activities. Besides, the roles of their parents and schools in equipping them with such knowledge should also be emphasized.

### 4.2. Students' Attitude towards NCSD Consumption

Regarding attitude towards NCSD consumption, about 50% of the students from either school accepted NCSD consumption. This may resulted from their low level of knowledge and lack of information about adverse effects of NCSD consumption on human health, The proportion of students who agreed and strongly agreed with NCSD consumption was over 60%. One noticeable result was that about one third of the students disagreed to stop drinking NCSD even though they were well aware of the health risks associated with consumption of NCSD. This unfavorable attitude is correlated with their low level of knowledge about NCSD. This result was quite consistent with that of a study on KAP of NCSD consumption conducted on 110 second-year medical students in India, in which 69.2% of students had tried to stop or quit the habit of consuming NCSD. This means just over 30% of them did not want to quit the habit of consuming such drinks [12].

### 4.3. Frequency and Level of Consumption

Ourstudy results showed that 100% of the students used to drink NCSD. The proportion of students consuming NCSD within one month prior to the study was very high (83.1%). This result indicated that NCSD were appealing to and commonly consumed among the study participants.

Students who drank NCSD 1–2 times/week accounted for the highest proportion (21.6%) while very few of them drank daily (8.4%). These results were some what incosistent with the results reported in a study by W&S, in which the proportions of participants who drank NCSD 3–4 times/week and 1–2 times/week were found highest, both standing at 28.6% [14]. This may be because out data collection was carried out in end-April to early summer when the level and amount of consumption were lower than in late summer. Indeed, people tend to consume more NCSD in August—the hottest month of the year, according to a study by W&S [14]. The frequency of consuming NCSD was high on occasions such as parties or family gatherings when NCSD as well as other foods and drinks are more likely to be consumed. However, the frequency of consuming NCSD in our study was lower than that in two studies in India and the USA [5],[13],[16].

On average, each student consumed 2,094 ml NCSD of all types (equivalent to six cans), or an annual average of 25 liters each month. Currently different levels of NCSD consumption among Vietnamese people have been reported. A report indicated that the total amount of NCSD in Vietnam in 2013 was estimated to be 927 million liters, equivalent to 10 l/person/year [14]. According to another report, the total amount of NCSD consumption continuously increased, and the national total NCSD consumption in 2013 was 2,083 million liters [15]. If that amount was divided by 90 million Vietnamese people, each individual would consume about 23 liters a year. The level of NCSD consumed by the students in our study (25 l/person/year) was higher than those in the two reports mentioned above. This can be explained by the fact that the two reports calculated the level of NCSD consumption per capita based on the amount of NCSD sold to all Vietnamese people, including young children, elderly people and those living in remote and secluded areas - those who tend to drink less and live in disadvantaged areas. Our study was, however, conducted in the capital city of Hanoi -the leading socio-economic and cultural center of the country where students can easily access and consume a higher amount of NCSD. Besides, the study's participants belonged to a group with the highest level of NCSD consumption [14],[15],[17].

However, the levels of consumption by Vietnamese people in general and by the students in our study were much lower than in certain studies conducted in other countries. For example, the total amount of NCSD consumed worldwide in 2012 was 220 billion liters per year. Each American consumed an annual average of about 43–46 liters as opposed to 23.1 liters by a Japanese person [8],[18].

### 4.4. Limitations

The students were asked about different types of NCSD that they had consumed within one month before the study and to estimate the amount they consumed per occasion. Although investigators gave hints to students to remind them of the occasions on which they might consume NCSD, such as birthday parties or when they participated in sport activities, etc recall bias might occur as the students might forgot some occasions. At the same time, although pictures of NCSD containers such as bottles, cans or glasses were shown to students in order to help them estimate the amounts of NCSD they used to consume, there by assisting investigators in converting those amounts into milimiters, information biases were inevitable.

This study referred to questions on knowledge and attitude as well as the semi-quantitative food frequency questionnaire from prior studies and adapted them to study participants and sites. Although the questionnaires had been piloted and revised before data were officially, another limitation of this study is that these quesitonnaires were not validated among Vietnamese young people.

## 5. Conclusion

Most students consumed NCSD within one month prior to the study, but the rate of students with daily consumption of NCSD was not high; the consumption of NCSD among suburban students was higher than that among urban ones, and male students consumed larger amount of NCSD than did female ones. Students from both school had limited knowledge of and unfavorable attitude towards NCSD. Therefore, communication and raising the awareness of students are necessary to reduce health risks when consuming NCSD. Families and schools shall be an important channel of communication and education in minimizing the consumption of NCSD. There is a need of further studies at alarger and deeper scale regarding NCSD consumption and health risks associated with NCSD consumption in the Vietnamese population.
